# Notes on Nervous and Mental Manifestations Due to Arteriosclerosis1Read at the forty-second annual meeting of the Medical Association of Central New York, held at Auburn, October 19, 1909.

**Published:** 1910-03

**Authors:** F. H. Stephenson

**Affiliations:** Syracuse, N. Y. 431 University Building


### Notes on Nervous and Mental Manifestations Due to Arteriosclerosis[1]

By F. H. STEPHENSON, M. D., Syracuse, N. Y.

WHEN nervous or mental symptoms in elderly people have advanced to the period where a physician is consulted it is usually found that the arterial changes, if these be the cause of the symptoms, are well developed as shown either in hightension, subtension or in hard, beady and tortuous bloodvessels. The heart is often enlarged and overworked. There are often evidences of nephritis, diabetis, generally lowered or enfeebled metabolism with consequent toxemia and debility. How unfortu-

1. Read at the forty-second annual meeting of the Medical Association of Central New York, held at Auburn, October 19, 1909.

nate that so much mischief is usually done before opportunity presents itself for us to delay these pathological changes.

Frequent urinary tests, heart examinations, and examinations with the sphygomanometer, with personal histories of all of our patients when reaching the fiftieth year, or often earlier, would doubtless reveal beginning changes which if treated early would give to many a more comfortable and prolonged or a retarded old age. We have learned that we may have marked arterial hardening without increased pressure, and viceversa, and that each leads to or may be a precursor of the other.

We know that generalised, not-localised sclerosis and very high or very low meter reading, with fair adjustment of all internal organs, often passes onward without uncomfortable symptoms. At other times a changed vascular condition, without compensation is present, yet patients remain quite free from suggestive symptoms. However, we must be on the lookout for trouble somewhere.

The blood pressure varies from different physical conditions, as digestion, exertion, periods of rest, relaxation, constipation, worry and freedom from toxic derangement, These variations are doubtless the great physical provisions for our protection from serious head or heart accidents.

We are also aware of socalled functional hypertension, resulting from acute pain, labor pains and many drugs. For example: digitalis, strychnia, ergot, atropine, camphor, adrenalin, caffene, lead poisoning, gouty and other toxins, nicotine and alcoholics. In persistent hightension, then, let us look for mischief, either in the heart, the bloodvessels themselves, the kidneys or in general metabolism, and these will usually develop some serious change in the nervous system even before a hemorrhage shall have taken place.

The mental symptoms of elderly people suffering from cardovascular changes are of the depressive type as melancholias, hypochondrias and monomanias, alternating with acute manic excitement, while others present a picture of dementia. Still others are easily excited, suspicious, irritable and emotional, keeping their family in a state of discomfort and unrest. Yet, again, others we find are markedly forgetful and unable to concentrate their minds. They suffer from headache and head pressure, they cry or laugh easily, suffer from insomnia, hissing and crackling sounds in the ears, have epileptoid seizures, vertigo, numbness with pitching sensations, extreme dizziness when turning in bed, an uneven gait, in some cases a tendency to go to the right or left, instead of advancing in a straight forward direction, depending upon the site of the sclerosis. Double vision and flashes of light before the eyes, exhaustion and general neurasthenic manifestations are often present.

Many of our cases have been treated for intercostal neuralgia and indigestion, when they really suffered from true angina, resulting from a sclerosis and spasm of the coronary arteries, or hardening or spasm of the bloodvessels of the stomach. They sometimes have slight aphasias, even when not preceded by any apparent symptoms of hemorrhage, but following fatigue. These attacks have been quite transient, passing away in a few hours. Sharp neuralgic head pains are frequent and are attributed to spasm of the temporal arteries. They also have prolonged periods of somnolence and sleep for twenty-four to forty-eight hours almost constantly. When they waken they are often dazed and disoriented.

In treating these conditions let us remember the four factors determining blood pressure as given by an able observer, whose name has escaped me. First, the action or energy of the heart; second, the peripheral resistance; third, the elasticity or rigidity of the vascular walls, fourth, the volume of the blood. These factors are most intimately coördinated by the nervous system. Careful hygienic living with moderate exercise, avoidance of worry, fatigue, over-work, over-eating and constipation, are the first prerequisites and prophylactics. The iodine preparations, the more recently discovered ones, specially sajodin, and iodalbin which are easily taken and well tolerated, I have found of marked value if long continued and at intervals increased in amount. These preparations reduce the viscosity of the blood, and favor elimination and absorption. Sodium silicate, 20 to 40 grains daily, is giving good results in cases with marked mental symptoms.

The nitrates and nitrites have their special use especially nitroglycerine in dilating the peripheral vessels and thus relieving internal congestion and pressure. They should be given frequently as their effect lasts only about forty-five minutes. Occasional doses of calomel, both in small and large does, followed by saline laxatives, give remarkable relief of the effects of hightension, possibly due in part to the diuretic and flushing effect on the kidneys and digestive tract. Theobromin, sodium salicylate, 5 grains and tincture of strophanthus, m. x. each, three times daily, have a most beneficial effect on the action of the heart and the vascular system, regulating the heart, lowering pressure and thus relieving cerebral pain, restlessness and fears.

High frequency electricity has given me most satisfactory results in a very large number of cases. I have employed it for the past three years very carefully, recording the pressure and pulse rate before and after treatment in quite critical cases with serious complications. I have many patients who are very enthusiastic over the benefits obtained from this treatment. Many of whom had received no benefit from various other methods. The tension has been reduced from 270 and higher to 130. Pulse rate, from 100 to 70 with restored rhythm; the head pressure, dizziness, vertigo, insomnia and general restlessness being relieved as well as digestion and general assimilation improved.

These treatments have been given daily or on alternate days for some weeks, followed by two treatments per week, then one, the length of administration varying from thirty to fifty minutes. I usually discontinue treatments after one or two months, resuming it later for a like period or longer. Some physicians are skeptical of the benefits of electricity in these cases, but after several years of daily treatment of these patients with differing nervous and mental symptoms, I am convinced that the benefit is more than psychical as is claimed by some. The condenser couch or chair I find most satisfactory for this work; it is comfortable, free from any pain or discomfort. In fact, the patient need scarcely feel it.

The D'Arsonval current from the apparatus made by Van Houten and Ten Broeck, taking the spark from the static machine, I employ and prefer it, though I have used the Hyfrex coil. The former necessitates giving the treatment in one's office, while the coil can be carried about and the current taken from the ordinary electric light burner. The lowered tension increased and equalised heat of the body, and the general well-being felt by the patient following the treatment, is most gratifying to him and very satisfying to the operator.

431 University Building.

